# Leveraging Artificial Intelligence to Inform Care Coordination by Identifying and Intervening in Patients' Unmet Social Needs: A Scoping Review

**DOI:** 10.1111/jan.16874

**Published:** 2025-03-10

**Authors:** Victoria H. Davis, Andrew D. Pinto, Minal R. Patel

**Affiliations:** ^1^ Department of Health Behavior & Health Equity University of Michigan School of Public Health Ann Arbor Michigan USA; ^2^ Upstream Lab, MAP Centre for Urban Health Solutions, Li ka Shing Knowledge Institute Unity Health Toronto Toronto Canada; ^3^ Department of Family and Community Medicine St. Michael's Hospital Toronto Canada; ^4^ Department of Family and Community Medicine Faculty of Medicine, University of Toronto Toronto Canada

**Keywords:** artificial intelligence, care coordination, case management, integrated care, machine learning, natural language processing, nursing, scoping review, social determinants of health, social needs

## Abstract

**Aim:**

We reviewed how artificial intelligence has been applied to inform care coordination by identifying and/or intervening in patients' unmet social needs.

**Design:**

Scoping review.

**Data Sources:**

PubMed, CINAHL, PsycInfo, and Scopus databases were searched for articles published by November 2023.

**Methods:**

Articles were excluded if they were reviews or protocols, did not explicitly mention artificial intelligence, or did not primarily focus on using it to identify and/or address unmet needs to inform care coordination.

**Results:**

Of 476 articles that underwent title and abstract screening, 102 were assessed for full‐text eligibility, and eight were ultimately included. Five articles used both natural language processing and machine learning; two articles used natural language processing; and one article used machine learning. Half (*n* = 4) of the articles focused on using artificial intelligence to identify/predict social needs, and two each focused on artificial intelligence to examine social resource provision or to indirectly identify social needs or using artificial intelligence to facilitate addressing unmet needs through care coordination.

**Conclusions:**

This review can inform an understanding of facilitators and barriers to the implementation of artificial intelligence in practice, to potentially improve patient care, health outcomes, and population health equity.

**Implications for Patients and the Profession:**

Using artificial intelligence to promote care coordination can expand opportunities to identify and intervene on social needs across more patients, with implications for nurses and other health professionals. It can also potentially exacerbate inequities and harm patient trust.

**Impact:**

The findings suggest a gap between the practice of incorporating artificial intelligence into integrated care platforms and the available scientific literature. This review can provide healthcare providers and organisations with insights into integrating artificial intelligence into clinical workflows, which may inform decisions about whether or how to implement these technologies in clinical settings.

**Reporting Method:**

We followed PRISMA‐ScR guidelines.

No Patient or Public Contribution.

**Summary**

- What is already known?
  ○ Care coordination can improve health outcomes, patient satisfaction, access to quality health services and decrease costs associated with hospital readmissions. The rising number of patients with multiple chronic comorbidities and social risks may impact the capacity of health systems and care coordinators to intervene in unmet social needs.
- What this paper adds
  ○ Our review highlights both the limited number of included articles and the innovative applications of artificial intelligence to inform social needs care coordination, including identifying or predicting unmet social needs, social resource provision, and facilitating interventions for unmet social needs.
- Implications for practice/policy
  ○ Using artificial intelligence to promote care coordination can expand opportunities to identify and intervene on social needs across more patients, and it can potentially exacerbate inequities and harm patient trust. This review can inform healthcare providers and policymakers about the barriers and facilitators to the implementation of artificial intelligence for social needs care coordination in practice.

## 1 Introduction

There is growing interest in leveraging innovative technologies to seamlessly predict and assist with patients' unmet social needs in healthcare. Social needs (e.g., social support, childcare, food, housing) are unmet when individuals do not have the non‐medical necessities to maintain a healthy life (Davis et al. 2023; Kreuter et al. 2021a). Unmet social needs are correlated with chronic diseases, stress, mental illness, substance use, fewer preventative care appointments, worse quality and access to care, and others (Kreuter et al. 2021b; Bisgaier and Rhodes 2011; Cole and Nguyen 2020). In the United States, the rising number of individuals living with multiple chronic diseases requires strong coordination between healthcare and social service providers (Martinez et al. 2019).

Care coordination includes the organisation of resources or personnel to improve the delivery of services and information relevant for high‐quality patient care (McDonald et al. 2007; Albertson et al. 2022). Care coordination requires communication and collaboration across providers, the patient, and their family, across and/or within settings, in alignment with meeting the priorities of the patient (Lamb 2014). While care coordination requires interdisciplinary collaboration, nurses, social workers, community health workers, and primary care providers are involved in providing different types and levels of assistance for patients with unmet social needs (Lamb 2014; Couturier et al. 2023; Doty et al. 2020). Nurses maintain an integral role in facilitating care coordination across multiple settings and have contributed to the practice of care coordination and coordination models (Lamb 2014). They are well positioned to contribute to the coordination of social care efforts from their nursing competencies, experiences, and role in patient‐centered care (Lamb 2014).

Care coordination can improve health outcomes and decrease costs associated with hospital readmissions (Berkowitz et al. 2018; Duminy et al. 2022), improve patient satisfaction, and increase access to quality health services (Baxter et al. 2018). For example, patients with unmet social needs may receive a referral from their primary care provider to a social worker, nurse, community health worker, system navigator, or other providers who offer knowledge related to care management and planning, resources for medical and non‐medical assistance, and connections to community service organisations (Albertson et al. 2022; Karam et al. 2021). However, care coordination requires a significant amount of training and knowledge of up‐to‐date resources and community services. This could potentially contribute to burnout among care coordinators and health professionals (Whitebird et al. 2017; Kung et al. 2019), who frequently have high caseloads of patients, responsibilities, and administrative tasks (Martinez et al. 2019; Schneider‐Kamp 2021). It can also be unclear how to focus social services on those with the greatest need or willingness to access such services, given resources, funding, and capacity constraints.

The use of artificial intelligence (AI) to facilitate cross‐sectoral services related to unmet social needs is a promising new avenue of research with relevance across countries with similar care coordination models and resources. AI is used for precision medicine, diagnostic imaging, disease surveillance and predicting future risk, remote digital visits, health diagnoses and treatment, and patient care assistance (Bohr and Memarzadeh 2020; Davenport and Kalakota 2019). The use of AI in these settings can potentially improve efficiency, accuracy, and decrease healthcare costs (Khanna et al. 2022).

Studies have recently used AI, particularly machine learning (ML) and natural language processing (NLP), to identify unmet social needs based on population or electronic health record (EHR) information (Patra et al. 2021; Han et al. 2022; Iacobelli et al. 2023). Using AI to derive or automate the collection of unmet social needs can help to inform opportunities to provide more robust care coordination. Further, adopting an EHR‐integrated AI database and risk stratification models can streamline assistance for social needs resources in a standardised manner (Kwon et al. 2021). For example, a study found that an AI algorithm trained on medical and social information provided tailored transitional care suggestions that, when implemented, helped to reduce rehospitalization by 21% among older patients compared to those who did not receive AI recommendations (Brown et al. 2023). Other methods may go further than solely identifying needs or patients, such as informing resource allocation or decision‐making regarding unmet social need assistance (Vest et al. 2019), or assistive AI technologies (e.g., chatbots) on the provider or patient side (Schario et al. 2022). The use of AI related to identifying or informing interventions for unmet social needs, such as resource allocation for social needs assistance, can have significant ethical consequences in perpetuating health inequities (Hewner et al. 2023; Obermeyer et al. 2019). Recent articles concentrate on the use of AI to identify or predict unmet social needs (Patra et al. 2021; Han et al. 2022). However, less attention has been given to the use of AI to inform care coordination specific to unmet social needs.

### 1.1 Key Terms

#### 1.1.1 Artificial Intelligence

AI is a set of tools that enable computers to perform analyses and make decisions in a manner similar to humans (Jiang et al. 2017).

#### 1.1.2 Care Coordination

Process of organising multiple resources or staff to improve the delivery of services and information relevant for high‐quality patient care (McDonald et al. 2007; Albertson et al. 2022). It can refer to coordination of patient care within the healthcare system, or between systems, including healthcare and community services (McDonald et al. 2007; Albertson et al. 2022). Care coordination requires communication and collaboration across providers, the patient and their family, across and/or within settings, in alignment with meeting the priorities of the patient (Lamb 2014).

#### 1.1.3 Machine Learning

A type of AI involving statistical and algorithmic techniques to facilitate computer learning to classify or predict patterns based on different levels of human supervision (Panch et al. 2018).

#### 1.1.4 Natural Language Processing

A type of AI that can identify, analyse, and/or create human language (Locke et al. 2021).

#### 1.1.5 Social Needs

Non‐medical financial and social necessities for healthy living (e.g., social support, childcare, food, housing) at the individual level, and are unmet when individuals do not have these necessities and cannot maintain a healthy life (Davis et al. 2023; Kreuter et al. 2021a).

## 2 The Review

### 2.1 Aim

This review aims to describe how AI is applied to inform care coordination by identifying and/or intervening in patients' unmet social needs. This information can help to assist with understanding barriers and facilitators to the implementation of AI to inform care coordination in practice, potentially impacting patient care, health outcomes, and population health equity.

### 2.2 Design

We decided to conduct a scoping review due to the emerging field of using AI to inform social needs care coordination. A scoping review methodology enabled a preliminary understanding of the research in this area, while incorporating a systematic search strategy (Grant and Booth 2009). We used the standard Preferred Reporting Items for Systematic reviews and Meta‐Analyses extension for Scoping Reviews (PRISMA‐ScR) guidelines to assist with organising and reporting the studies (Tricco et al. 2018).

### 2.3 Search Methods

A preliminary exploration of the available literature was first conducted to identify relevant search terms to guide the review. A trained information specialist was consulted to assist with the preliminary search strategy, terms, and database selection. We searched PubMed, CINAHL, APA PsycInfo, and Scopus databases for studies published up to November 2023, with no prespecified start date. Our search strategy incorporated terms for care coordination (e.g., case management, integrated care, and patient navigation), AI (e.g., machine learning, neural networks, and large language models), and social needs or social determinants of health (e.g., financial stress, food security; Data S1: Search Strategy).

### 2.4 Inclusion and Exclusion Criteria

The inclusion criteria encompassed English‐language, peer‐reviewed articles that focused on using AI to inform care coordination by identifying and/or intervening in patients' unmet social needs. We did not include a particular time period or parameters related to country, and the population of interest was broadly focused on patients in health settings (e.g., inpatient and outpatient care) to provide an overview of populations described on this topic. Articles were excluded if they were reviews or study protocols, did not explicitly mention AI technologies (e.g., deep learning, natural language processing, support vector machines, ML), or did not primarily focus on using AI to inform care coordination for the purpose of identifying unmet social needs and/or addressing unmet social needs. Articles that used AI to predict unmet social needs for the *sole* purpose of medical or healthcare utilisation outcomes were not included. For example, articles that focused only on predicting inpatient hospitalisations or mortality and morbidity from a particular condition were excluded. Articles that only used AI as an analysis method (e.g., regression models) were not included as they were not *about* AI.

### 2.5 Search Outcome

**FIGURE 1:**
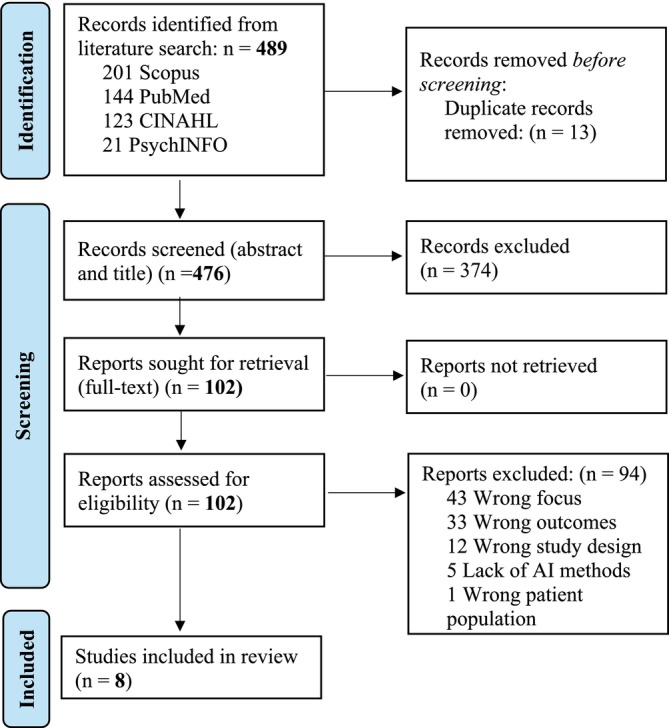
Adapted PRISMA‐ScR flow diagram (Tricco et al. 2018).

A total of 489 articles were identified from the searches. After the removal of duplicate articles, 476 articles underwent title and abstract screening. There were 102 articles that were assessed for full‐text eligibility, and eight were ultimately included (see Figure 1. PRISMA Diagram) (Tricco et al. 2018).

### 2.6 Quality Appraisal

We did not conduct a quality appraisal of the articles. We excluded certain types of articles (protocols and reviews) and included peer‐reviewed articles to assist with a robust description of the included studies.

### 2.7 Data Abstraction

Search results were exported from each of the included databases and imported into the Zotero citation manager, followed by Covidence for duplicate removal and level 1 and level 2 article screening. A single reviewer was engaged in screening and article selection. A standardised data extraction form was created in Microsoft Excel to capture the relevant information, including authors, country, participant sample, study design, data collection method, AI used, description of social needs captured, and main findings.

### 2.8 Synthesis

Given the paucity of peer‐reviewed literature on this topic, we provided a rich description of the included studies. Upon reading the included articles in depth, we categorised the studies based on the purpose of using AI to inform care coordination in relation to unmet social needs. There were three main purposes: (1) AI to identify or predict unmet social needs, (2) AI to examine social resource provision or indirect identification of unmet social needs, and (3) AI to facilitate addressing unmet social needs through care coordination, integration, or care management.

## 3 Results

### 3.1 Overview

All studies were based in the United States. Included articles were retrospective analysis/chart reviews (*n* = 5), case studies (*n* = 2), and a secondary analysis of a longitudinal study (*n* = 1). The studies reported variable social needs, with some studies focusing on specific needs (e.g., three articles focused primarily on housing instability or homelessness) and the remaining having non‐specific social needs. Five articles used both ML and NLP; two articles used NLP; and one article used ML. Simplified characteristics of the studies are in Table 1 (see Data S2 for more study details).

**TABLE 1:** Characteristics of included studies (*n* = 8).

Lead author name, date	Aim of study	Study design	Type of AI	Description of AI used	Type of social needs assessed or addressed
Snowdon et al. 2020	To explore the implementation, processes, and effects of an integrated data system and hub for care coordination of government resources.	Case study	NLP, ML	IBM Watson Care Manager (WCM) is an integrated, coordinated care platform. It aggregates and coordinates information from community organisations and health and social care providers. Extracts information on patient social needs.	Focused largely on unhoused individuals with other complex needs. Care coordination assisted with finances, employment, food, housing, substance use, disability, behavioural health.
Hewner et al. 2023	To explore the contribution of nursing knowledge to an unsupervised ML classification model using psychosocial phenotyping to segment patients based on medical, behavioural, and social need factors.	Retrospective analysis/chart review	ML	Unsupervised ML k‐means clustering using elbow‐method	Social factors (age, sex, childhood illness, ethnicity, language, race, refugee status), behavioural health factors (substance use, mental health).
Iacobelli et al. 2023	To assess different ML algorithms to predict social determinants of health, and whether solely demographic information or demographic information and navigation notes assist with prediction of the larger determinants.	Retrospective analysis/chart review	NLP, ML	Experiment 1: ML (logistic regression, random forest, support vector machine, artificial neural network, Gaussian naive Bayes); Used NLP (latent Dirichlet allocation—LDA) for free‐text notes. Experiment 2: multilabel classification model (convolutional neural network).	A broad range of 22 social determinants of health categories (see page 5).
Schario et al. 2022	To explore nurse care management experiences with chatbots and provide insight on its implementation.	Case study	NLP	AI chatbot. Care manager receives alerts and documentation of chatbot conversations when needed, to assist the care manager with providing tailored intervention based on their needs.	Mentions generally that social determinants of health can be better addressed through communication with the chatbot. Discussed education, referrals to social work, tobacco cessation education, and others.
Bako et al. 2021	To parse and classify events where social workers addressed patients' unmet social needs using ML and NLP.	Retrospective analysis	NLP, ML	NLP, ML/deep learning to automatically classify events where social workers intervened on patients' unmet social needs. Tested multiple algorithms: rule‐based, logistic regression, kernelized and linear support vector machine, and Multinomial Naive Bayes	Classification scheme of social work interventions included financial planning, supportive counselling, care coordination, education, community service, application filing and reporting, housing, transportation, medical equipment, or legal services.
Bako et al. 2020	To identify the unmet social needs of patients referred to social work care.	Analysis of a longitudinal cohort study	NLP, ML	NLP and market basket analysis (to understand the order of unmet social needs that led to a referral to a social worker).	17 social needs and health categories, e.g., financial, food, safety, housing, legal, language services, community resources.
Gray et al. 2023	To create and evaluate an NLP and text mining model to characterise residential instability, transportation needs, and food insecurity from EHR data.	Retrospective analysis/chart review	NLP, ML	NLP and text mining. Rule‐based and deep learning ML models (note‐based, terminology‐based, and ClinicalBERT).	Residential instability, food insecurity, transportation challenges
Hatef et al. 2022	To examine the performance of an NLP algorithm on identifying residential instability across 3 health systems.	Retrospective analysis/chart review	NLP	Rule‐based NLP system that was informed by experts in medicine and residential instability.	Residential instability (homelessness and housing instability)

Multiple AI approaches were discussed. Articles centred on the use of NLP to extract and identify free‐text notes from the EHR and used ML to segment or cluster patients based on a combination of clinical, psychosocial, and behavioural characteristics. The studies can be categorised based on the application of AI to inform care coordination of unmet social needs, including (1) to identify/predict unmet social needs; (2) examine social resource provision or indirect identification of unmet social needs; and (3) to facilitate addressing unmet social needs through care coordination, integration, or care management.

### 3.2 AI to Identify or Predict Unmet Social Needs (*n* = 4)

Half (*n* = 4 of 8) of the articles described using a combination of ML and NLP to inform the identification and classification of unmet social needs from existing data. Two articles examined NLP rule‐based algorithms to identify or categorise patients by social need (Gray et al. 2023; Hatef et al. 2022). For example, one article created and evaluated an NLP rule‐based algorithm to identify residential instability, transportation challenges, and food insecurity in comparison to ML algorithms in an integrated healthcare system (Gray et al. 2023). Similarly, another article assessed the performance of an NLP rule‐based algorithm in identifying residential instability across three different integrated healthcare delivery systems and had modest precision (Hatef et al. 2022). For feature development, these articles used manual and semiautomated lexicon curation (Gray et al. 2023; Hatef et al. 2022).

One study included unsupervised ML k‐means clustering using an elbow method to group high‐needs patients (Hewner et al. 2023). Another study examined various ML algorithms in addition to NLP for free‐text notes in their ability to predict unmet social needs and assessed whether providing solely demographic information or combining that information with patient navigation notes improved the performance of the model (Iacobelli et al. 2023).

Three articles incorporated similar levels of input from experts to inform their models (Hewner et al. 2023; Gray et al. 2023; Hatef et al. 2022). For example, two studies incorporated expert‐informed knowledge related to defining key social needs and terms to include in their NLP model (Gray et al. 2023; Hatef et al. 2022). Similarly, one article incorporated the knowledge of three expert nurses to inform their unsupervised k‐means cluster analysis (Hewner et al. 2023). Specifically, nurses chose the variables that would subsequently be used in an unsupervised k‐means cluster analysis using the elbow method, and their input informed the psychosocial phenotyping of patients based on their clinical knowledge of high‐needs cases (Hewner et al. 2023).

### 3.3 AI to Examine Social Resource Provision or Indirect Identification of Unmet Social Needs (*n* = 2)

Two studies by (Bako et al. 2021, 2020) from Indiana University specifically examined social work referrals to address unmet social needs using AI (Bako et al. 2021, 2020). One article described using NLP to extract and retrieve unstructured text information (e.g., clinical notes) of social work interventions from the EHR in a federally qualified health center (Bako et al. 2021). The authors also used rule‐based algorithms, ML algorithms, and a deep learning long short‐term memory recurrent neural network for multilabel classification of social work interventions (Bako et al. 2021). The second study used NLP and market basket analysis to detect the unmet social needs among patients who were given a referral to social work and the co‐occurring unmet social needs to inform future social work resource provision (Bako et al. 2020). The authors found that 22% of all patients at the safety net organisation were given a social work referral, many of whom were female, younger, and Hispanic and had multiple comorbidities (Bako et al. 2020). Social work interventions were most common for financial needs or pregnancy‐related needs (25% of referrals for each), and 7% of patients had co‐occurring pregnancy needs and English language difficulties (Bako et al. 2020).

### 3.4 AI to Facilitate Addressing Unmet Social Needs Through Care Coordination, Integration, or Care Management (*n* = 2)

Two of the eight articles examined ways that AI can be used to foster social resources to address unmet social needs through the integration of adapted technology during care coordination or care management (Schario et al. 2022; Snowdon et al. 2020). One case study described the implementation of a care coordination chatbot that uses NLP, specifically, large language models, to provide a conversation interface directly to patients at high risk of hospital readmission and with chronic diseases that may be more difficult to reach through coordinated care and care management (Schario et al. 2022). The care manager receives alerts based on the patients' conversations with the chatbot and uses this information to speak directly with the patient prior to attempts to address unmet social needs through various interventions and social work service referrals (Schario et al. 2022).

Another case study describes IBM Watson Care Manager (now Merative Integrated Care), which is a cloud‐based care management and coordination platform that provides interoperable, multidisciplinary, and team‐based communication to facilitate patient care across a variety of community, government, social, and medical providers (Snowdon et al. 2020). The platform assists with scheduling, tailored patient care planning, and communication across providers of different types to improve care (Snowdon et al. 2020). The system uses a combination of NLP and ML approaches and both identifies patients' unmet social needs through a variety of texts included in the platform as well as facilitates referrals to intervene in patients' unmet social needs (Snowdon et al. 2020). The article describes how this platform was implemented during the deadly wildfires in California that displaced many individuals from their homes and the influx of individuals who identified as unhoused or housing‐limited (Snowdon et al. 2020). The platform was used to provide services to 77 individuals with high care needs (Snowdon et al. 2020).

## 4 Discussion

This scoping review explored how peer‐reviewed literature has applied AI to inform care coordination for unmet social needs. Overall, the review found few studies that met the inclusion criteria, and the included studies were published between 2020 and 2023. This suggests that this topic is a new, emerging area, and the review provides a preliminary snapshot of the potential for a highly varied range of AI tools to assist with care coordination. A disproportionately greater number of studies (*n* = 6 of 8) focused solely on the application of AI to either identify or predict unmet social needs using ML and NLP algorithms, followed by using AI to examine social resource provision or indirect identification of unmet social needs (*n* = 2 of 8) and to facilitate intervening in unmet social needs through care coordination, integration, or care management (*n* = 2 of 8).

Only two of the six articles focusing on the identification of unmet social needs or resource provision explicitly included methods that consider the co‐occurrence of unmet social needs or provide consideration for the importance of intersectionality (Iacobelli et al. 2023; Bako et al. 2020). In other words, most of these articles portray unmet social needs as existing in a vacuum, presenting singularly and in the same way for everyone (Iacobelli et al. 2023). This is important because perceived individual identities impact opportunities, treatment, care, and potential discrimination within the healthcare system in a complex manner. Implementing algorithms that do not *explicitly* consider or account for structural inequities, resources, and differentially intersecting disadvantage or advantage through social needs may contribute to inadequate, wasteful, or harmful care. While AI can have the potential to improve clinical operations, patient care, and health equity, there could be a tendency to forget that patients living with unmet social needs are more than a data point. Patients should be considered and partnered with at every stage of discussion about implementing AI to identify and/or intervene in unmet social needs.

Importantly, many applications of AI in healthcare settings contribute to perpetuating or sustaining racial and healthcare disparities through biased data and algorithms. In particular, the use of AI to assist with decision‐making surrounding resource allocation to individuals can be extremely dangerous, particularly if healthcare utilisation or cost‐related predictors are included in the algorithm (Hewner et al. 2023; Obermeyer et al. 2019). This is because differences in healthcare utilisation or cost, for example, often reflect larger structural and racial disparities related to access, racism, and other inequities (Hewner et al. 2023; Obermeyer et al. 2019). However, only two of the included articles explicitly highlighted the dangers of using AI to identify or inform social care to patients (Iacobelli et al. 2023; Hewner et al. 2023). Hewner et al. (2023) incorporated EHR data alongside expert nurse perspectives to create clusters of patient phenotypes and increase the “real‐world” clinical application of their ML algorithm, while acknowledging that their final algorithm will not include cost‐related predictors. This approach does not acknowledge how expert providers may themselves have biased views that, when incorporated into algorithms, can contribute to health inequities (Schario et al. 2022; Snowdon et al. 2020).

Overall, there was variability across studies in the particular AI analysis or approach, the performance of the included algorithms, the social needs categories discussed, the level of detail provided on the AI algorithms and study sample, and the study quality given the high number of case studies and chart reviews. For example, of the two studies that focused on using AI to address unmet social needs through care coordination or management, the IBM Watson Care Manager (now Merative Integrated Care) presents an innovative integrated care coordination system across health, social, and government agencies through the proprietary IBM software (Snowdon et al. 2020). However, other than indicating that 77 individuals were impacted, it is unclear exactly what that means and whether this software has clinically meaningful outcomes. The proprietary commercial ownership of many of the existing care coordination AI platforms may contribute to the lack of peer‐reviewed literature on these platforms. This poses a challenge to robustly assessing and investigating biases and subsequent impact on patient care and outcomes. Further, it is possible that care providers, after becoming acquainted with care coordination platforms, unquestionably adopt them and trust such platforms while not understanding the details of how the algorithms work. For example, there are multiple companies that focus on using AI to address unmet social needs through care coordination that are currently implemented by major healthcare systems, such as WellSky Social Care Coordination (Social Care Coordination n.d.), naviHealth Community platform (naviHealth 2022), Healthful (Businesswire 2021), and more.

### 4.1 Future Directions

Together, the findings suggest a significant gap between the practice of incorporating AI to inform the identification of unmet social needs and more connected, integrated care platforms, and the scientific literature describing the applications of these methods. This has important implications: (1) there is a lack of information on the acceptability and feasibility of adopting these methods into clinical care, as well as their utility, from the perspectives of a range of health and social care providers, administrators; and (2) understanding from patients whether these targeted social need resources are impactful for them “in the real world” and assist with greater accessibility and availability of resources, as opposed to perpetuating harms, including racial discrimination. To advance the field of AI and facilitate health and healthcare equity, future work should conduct research on the perspectives of providers, patients, and other key groups, particularly regarding the implementation of AI to provide coordinated, integrated care that addresses unmet social needs.

### 4.2 Strengths and Limitations

As with all studies, this review has multiple limitations. First, while this study presents an important overview of the topic discussed, it lacks a systematic approach to the selection of studies (e.g., having two independent reviewers screen each article). Thus, there may be some articles missing, potentially contributing to the risk of bias in the review. However, aligned with the strengths of this study, efforts were made to mitigate bias, including consultation with a professional information specialist for preliminary guidance on the search terms and search strategy across four major databases. This has the potential to improve the rigor and sensitivity of the search.

## 5 Conclusions

This review found very few studies that apply AI to inform care coordination by identifying unmet social needs, and in particular, to intervene in unmet social needs through care coordination or management. This work could inform discussions among patients, decision‐makers, social care, and healthcare providers regarding existing opportunities to implement AI in this area and the risks in doing so. Research and policy efforts should explore avenues to address community and patient concerns and mitigate ethical issues surrounding the use of AI in care coordination, with a focus on its potential to exacerbate health inequities and patient safety.

## Author Contributions

**Victoria H. Davis:** conceptualisation, design, analysis and interpretation of data, and manuscript writing (first draft and revisions). **Andrew D. Pinto:** analysis and interpretation of data and manuscript writing. **Minal R. Patel:** design, analysis and interpretation, manuscript writing. All authors have given their final approval of the manuscript and agree to be accountable for the work.

## Conflicts of Interest

The authors declare no conflicts of interest.

## Supporting information


Data S1.

Data S2.

## Data Availability

The data that support the findings of this study are openly available.
