# Supplementary material for: Leveraging Artificial Intelligence to Inform Care Coordination by Identifying and Intervening in Patients' Unmet Social Needs: A Scoping Review
Source: J Adv Nurs. 2025 Mar 10;81(12):8504–12. doi: 10.1111/jan.16874 (PMC12623701; doi:10.1111/jan.16874)
Supplement: Supplementary file 1 — Data S1. [file JAN-81-8504-s002.docx]

All searches were conducted on November 3, 2023.

1. **PubMed**
2. (("Community Support"[Mesh] OR "Case Management"[Mesh] OR "Transitional Care"[Mesh] OR "Patient Discharge"[Mesh] OR "Hospital to Home Transition"[Mesh] OR "Patient Handoff"[Mesh] OR "care coordinat*"[tiab] OR "Care transition*"[tiab] OR "Health navigat*"[tiab] OR "Case manage*"[tiab] OR "Clinical navigation"[tiab] OR "patient navigat*"[tiab] OR "Integrated care"[tiab] OR "Social care"[tiab])

AND

1. ("Artificial Intelligence"[Mesh] OR NLP[tiab] OR LLM[tiab] OR "Artificial intelligence"[tiab] OR "artificial general intelligence"[tiab] OR "artificial social intelligence"[tiab] OR "Machine Learning" OR "Natural Language Processing" OR "Natural language understanding" OR "computer neural networks" OR "Deep Learning" OR "Sentiment Analys*" OR "artificial neural network" OR "artificial narrow intelligence" OR "augmented intelligence" OR "intelligence augment" OR "optical character recognition" OR "cognitive comput" OR "recurrent neural network" OR "deep neural network" OR "naive bayes" OR "support vector" OR "random forest" OR "large language model" OR "Heuristic" OR "machine intelligence"[tiab:~2] OR "ChatGPT" OR "generative AI" OR "chatbot" OR "conversational AI" OR "robotic process automation" OR "AI healthcare" OR "AI health care" OR "medical AI" OR "AI application" OR "AI apps" OR "AI app" OR GPT[tiab] OR "generative pre-trained transformer" OR "generative pretrained transformer"[tiab]))

AND

1. ("child poverty"[majr] OR "education"[majr] OR "educational status"[majr] OR "employment"[majr] OR "food insecurity"[majr] OR "health equity"[majr] OR "health services accessibility"[majr] OR "health status disparities"[majr] OR "healthcare disparities"[majr] OR "income"[majr] OR "insurance"[majr] OR "low socioeconomic status"[majr] OR "medically uninsured"[majr] OR "poverty areas"[majr] OR "poverty"[majr] OR "psychosocial support systems"[majr] OR "social class"[majr] OR "social conditions"[majr] OR "social determinants of health"[majr] OR "social environment"[majr] OR "social justice"[majr] OR "social support"[majr] OR "socioeconomic disparities in health"[majr] OR "socioeconomic factors"[majr] OR "sociological factors"[majr] OR "unemployment"[majr] OR "working poor"[majr] OR "education level*"[tiab] OR "education status"[tiab] OR "educational achievement*"[tiab] OR "food insecur*"[tiab] OR "housing instability"[tiab] OR "medical bill*"[tiab] OR "psycho social"[tiab] OR "social class"[tiab] OR "social determinant*"[tiab] OR "social environment"[tiab] OR "social support"[tiab] OR "transportation barrier*"[tiab] OR accessibility[tiab] OR built environment [tiab] OR debt[tiab] OR disparit*[tiab] OR disparity[tiab] OR employ*[tiab] OR equalit*[tiab] OR equit*[tiab] OR financial[tiab] OR homeless*[tiab] OR income[tiab] OR inequalit*[tiab] OR inequit*[tiab] OR insuran*[tiab] OR insured[tiab] OR literacy[tiab] OR literate[tiab] OR neighborhood[tiab] OR poverty[tiab] OR psychosocial[tiab] OR sdoh[tiab] OR ses[tiab] OR sociodemographic[tiab] OR socioeconomic[tiab] OR underserved[tiab] OR unemploy*[tiab] OR unhoused[tiab] OR uninsured[tiab] OR "social need*"[tiab] OR "social risk"[tiab])

144 results

1. **APA PsychInfo**
2. (MH "Case Management+") OR (MH "Transitional Care") OR (MH "Multidisciplinary Care Team+") OR (MH "Transfer Techniques") OR (MM "Case Managers+") OR (MH "Community Health Workers") OR (MH "Health Care Delivery, Integrated") OR (MH "Patient Navigation") OR (MH "Community Service") OR (MH "Community Resources") OR (MH "Health Resource Allocation") OR (MH "Referral and Consultation") OR "care coordinat*" OR "Care transition*" OR "Health navigat*" OR "Case manage*" OR "Clinical navigation" OR “patient navigat*” OR "integrated care" OR "coordinat*" OR "social care"

AND

1. TI (MH "Artificial Intelligence") OR (MH "Machine Learning+") OR (MH "Natural Language Processing") OR (MH "Neural Networks (Computer)") OR NLP OR LLM OR "Artificial intelligence" OR “artificial general intelligence” OR “artificial social intelligence” OR "Machine Learning" OR "Natural Language Processing" OR “Natural language understanding” OR "computer neural networks" OR "Deep Learning" OR "Sentiment Analys*" OR "artificial neural network" OR “artificial narrow intelligence” OR “augmented intelligence” OR “intelligence augment” OR “optical character recognition” OR “cognitive comput*” OR “recurrent neural network” OR “deep neural network” OR "naive bayes" OR "support vector" OR "random forest" OR "large language model" OR "Heuristic" OR "machine intelligence" OR “generative AI” OR “chatbot” OR “conversational AI” OR “AI healthcare” OR “AI health care” OR “medical AI” OR “AI application” OR “AI apps” OR “AI app” OR GPT OR “generative pre-trained transformer” OR “generative pretrained transformer”

AND

1. (MH "Food Security") OR (MH "Debt, Financial") OR (MH "Economic Aspects of Illness") OR (MH "Economic and Social Security") OR (MH "Food Assistance") OR (MH "Insurance, Health") OR (MH "Financial Stress") OR (MH "Financial Management") OR (MH "Social Determinants of Health") OR (MH "Socioeconomic Disparities in Health") OR (MH "Nutritional Status") OR (MH "Psychosocial Functioning") OR (MH "Low Socioeconomic Status") OR (MH "Support, Social+") OR (MH "Work Environment") OR (MH "Social Isolation+") OR (MH "Homelessness") OR (MH "Poverty") OR (MH "Sociodemographic Factors") OR (MH "Socioeconomic Factors+") OR (MH "Social Class+") OR (MH "Social Environment") OR (MH "Psychosocial Deprivation") OR (MH "Healthcare Disparities") OR "child poverty" OR "education level*" OR "education status" OR "education" OR "educational achievement*" OR "educational status" OR "employment" OR "food insecur*" OR "food insecurity" OR "health equity" OR "health services accessibility" OR "health status disparities" OR "healthcare disparities" OR "housing instability" OR "income" OR "insurance" OR "low socioeconomic status" OR "medical bill*" OR "medically uninsured" OR "poverty areas" OR "poverty" OR "psycho social" OR "psychosocial support systems" OR "social class" OR "social conditions" OR "social determinant*" OR "social determinants of health" OR "social environment" OR "social support" OR "socioeconomic disparities in health" OR "socioeconomic factors" OR "sociological factors" OR "transportation barrier*" OR "unemployment" OR "working poor" OR accessibility OR built environment OR debt OR disparit* OR employ* OR equalit* OR equit* OR financial OR homeless* OR income OR inequalit* OR inequit* OR insuran* OR insured OR literacy OR literate OR neighborhood OR poverty OR psychosocial OR sdoh OR ses OR sociodemographic OR socioeconomic OR underserved OR unemploy* OR unhoused OR uninsured OR “social need*” OR "social risk"

Results: 32; Limited to academic journals = 21 total

1. **CINAHL**
2. (MH "Case Management+") OR (MH "Transitional Care") OR (MH "Multidisciplinary Care Team+") OR (MH "Transfer Techniques") OR (MM "Case Managers+") OR (MH "Community Health Workers") OR (MH "Health Care Delivery, Integrated") OR (MH "Patient Navigation") OR (MH "Community Service") OR (MH "Community Resources") OR (MH "Health Resource Allocation") OR (MH "Referral and Consultation") OR "care coordinat*" OR "Care transition*" OR "Health navigat*" OR "Case manage*" OR "Clinical navigation" OR “patient navigat*” OR "integrated care" OR "coordinat*" OR "social care"

AND

1. TI (MH "Artificial Intelligence") OR (MH "Machine Learning+") OR (MH "Natural Language Processing") OR (MH "Neural Networks (Computer)") OR NLP OR LLM OR "Artificial intelligence" OR “artificial general intelligence” OR “artificial social intelligence” OR "Machine Learning" OR "Natural Language Processing" OR “Natural language understanding” OR "computer neural networks" OR "Deep Learning" OR "Sentiment Analys*" OR "artificial neural network" OR “artificial narrow intelligence” OR “augmented intelligence” OR “intelligence augment” OR “optical character recognition” OR “cognitive comput*” OR “recurrent neural network” OR “deep neural network” OR "naive bayes" OR "support vector" OR "random forest" OR "large language model" OR "Heuristic" OR "machine intelligence" OR “generative AI” OR “chatbot” OR “conversational AI” OR “AI healthcare” OR “AI health care” OR “medical AI” OR “AI application” OR “AI apps” OR “AI app” OR GPT OR “generative pre-trained transformer” OR “generative pretrained transformer”

AND

1. (MH "Food Security") OR (MH "Debt, Financial") OR (MH "Economic Aspects of Illness") OR (MH "Economic and Social Security") OR (MH "Food Assistance") OR (MH "Insurance, Health") OR (MH "Financial Stress") OR (MH "Financial Management") OR (MH "Social Determinants of Health") OR (MH "Socioeconomic Disparities in Health") OR (MH "Nutritional Status") OR (MH "Psychosocial Functioning") OR (MH "Low Socioeconomic Status") OR (MH "Support, Social+") OR (MH "Work Environment") OR (MH "Social Isolation+") OR (MH "Homelessness") OR (MH "Poverty") OR (MH "Sociodemographic Factors") OR (MH "Socioeconomic Factors+") OR (MH "Social Class+") OR (MH "Social Environment") OR (MH "Psychosocial Deprivation") OR (MH "Healthcare Disparities") OR "child poverty" OR "education level*" OR "education status" OR "education" OR "educational achievement*" OR "educational status" OR "employment" OR "food insecur*" OR "food insecurity" OR "health equity" OR "health services accessibility" OR "health status disparities" OR "healthcare disparities" OR "housing instability" OR "income" OR "insurance" OR "low socioeconomic status" OR "medical bill*" OR "medically uninsured" OR "poverty areas" OR "poverty" OR "psycho social" OR "psychosocial support systems" OR "social class" OR "social conditions" OR "social determinant*" OR "social determinants of health" OR "social environment" OR "social support" OR "socioeconomic disparities in health" OR "socioeconomic factors" OR "sociological factors" OR "transportation barrier*" OR "unemployment" OR "working poor" OR accessibility OR built environment OR debt OR disparit* OR employ* OR equalit* OR equit* OR financial OR homeless* OR income OR inequalit* OR inequit* OR insuran* OR insured OR literacy OR literate OR neighborhood OR poverty OR psychosocial OR sdoh OR ses OR sociodemographic OR socioeconomic OR underserved OR unemploy* OR unhoused OR uninsured OR “social need*” OR "social risk"

Results: 146; limited to academic journals = 123

1. **Scopus**
2. ( TITLE ( "care coordinat*" OR "Care transition*" OR "Health navigat*" OR "Case manage*" OR "Clinical navigation" OR "patient navigat*" OR "community resource*" OR "referral" OR "integrated care" OR "integrat*" OR "social care" OR "community health" OR "social work*" )

AND

1. TITLE ( nlp OR llm OR "Artificial intelligence" OR "artificial general intelligence" OR "artificial social intelligence" OR "Machine Learning" OR "Natural Language Processing" OR "Natural language understanding" OR "computer neural networks" OR "Deep Learning" OR "Sentiment Analys*" OR "artificial neural network" OR "artificial narrow intelligence" OR "augmented intelligence" OR "intelligence augment" OR "optical character recognition" OR "cognitive comput*" OR "recurrent neural network" OR "deep neural network" OR "naive bayes" OR "support vector" OR "random forest" OR "large language model" OR "Heuristic" OR "machine intelligence" OR "generative AI" OR "chatbot" OR "conversational AI" OR "AI healthcare" OR "AI health care" OR "medical AI" OR "AI application" OR "AI apps" OR "AI app" OR gpt OR "generative pre-trained transformer" OR "generative pretrained transformer" )

AND

1. TITLE-ABS-KEY ( "child poverty" OR "education level*" OR "education status" OR "education" OR "educational achievement*" OR "educational status" OR "employment" OR "food insecur*" OR "food insecurity" OR "health equity" OR "health services accessibility" OR "health status disparities" OR "healthcare disparities" OR "housing instability" OR "income" OR "insurance" OR "low socioeconomic status" OR "medical bill*" OR "medically uninsured" OR "poverty areas" OR "poverty" OR "psycho social" OR "psychosocial support systems" OR "social class" OR "social conditions" OR "social determinant*" OR "social determinants of health" OR "social environment" OR "social support" OR "socioeconomic disparities in health" OR "socioeconomic factors" OR "sociological factors" OR "transportation barrier*" OR "unemployment" OR "working poor" OR accessibility OR built AND environment OR debt OR disparit* OR employ* OR equalit* OR equit* OR financial OR homeless* OR income OR inequalit* OR inequit* OR insuran* OR insured OR literacy OR literate OR neighborhood OR poverty OR psychosocial OR sdoh OR ses OR sociodemographic OR socioeconomic OR underserved OR unemploy* OR unhoused OR uninsured OR "social need*" OR "social risk* " )
