# Supplementary material for: Leveraging Artificial Intelligence to Inform Care Coordination by Identifying and Intervening in Patients' Unmet Social Needs: A Scoping Review
Source: J Adv Nurs. 2025 Mar 10;81(12):8504–12. doi: 10.1111/jan.16874 (PMC12623701; doi:10.1111/jan.16874)
Supplement: Supplementary file 2 — Data S2. [file JAN-81-8504-s001.docx]

| Lead author name, date | Country | Aim | Data collection method | Setting | Design | AI Application | AI | Description of AI | Type of social needs assessed or addressed | Sample | Main findings |
| --- | --- | --- | --- | --- | --- | --- | --- | --- | --- | --- | --- |
| Snowdon et al., 2020 | U.S. | To explore implementation, processes, and effects of an integrated data system and hub for care coordination of government resources. | Over 91,000 records were included from multiple resources (CalWIN, SWITS, Avatar, and IJS). | Sonoma County, California | Case study | Addressing unmet social needs through care coordination, integration, or care management. | NLP, ML | IBM Watson Care Manager (WCM) is an integrated care, HIPAA coordination platform. It aggregates and coordinates information from community organizations and health and social care providers. WCM contributes to scheduling, care plans, budgets, provider connections. WCM extracts information on patient health and social needs by examining care documentation. | Focus on unhoused individuals with complex needs. Indicated that care coordination assists with financial security, employment, food, housing, substance use, disability, and behavioural health. | 77 individuals at-risk of crisis during wildfires and with complex care and social needs. | Individuals with complex care needs were assisted after the 2017 Sonoma County wildfires through coordinated care and access to a range of services. |
| Hewner et al., 2023 | U.S. | Explore the contribution of nursing knowledge to the methods and initial results of an unsupervised ML classification model using psychosocial phenotyping to segment patients based on medical, behavioural, and social needs. | Primary care practice data | FQHCs in five urban areas with lower resource opportunities in Buffalo and New York. | Retrospective analysis/ chart review | Identify or predict unmet social needs | ML | Unsupervised ML k-means clustering using elbow-method | Non-specific; mentions social factors (age, sex, childhood illness, ethnicity, language, race, refugee status), behavioural health factors (substance use, mental health). | The "enhanced model" included 1,233 individuals living with diabetes that were identified as “high-needs” (at least 2 conditions) | After k-means clustering and incorporating nursing expertise, clusters were categorized into four psychosocial phenotypes: 1) large racially diverse female patients who do not speak English, have experienced childhood health issues, and are not medically complex; 2) large cluster of English language speakers with comorbidities; 3) small cluster of males living with substance use challenges, mental illness, chronic disease comorbidities and high hospital utilization; 4) medium-size cluster composed of racially diverse individuals who are of older age and experience renal failure. |
| Iacobelli et al., 2023 | U.S. | Assess different ML algorithms to predict SDoH, and whether solely demographic information or demographic information and navigation notes assist with prediction. | Patient navigator notes and demographic information. | Chicago (Chinatown and DuPage County) | Retrospective analysis/ chart review | Identify or predict unmet social needs | NLP, ML | Experiment 1: ML (logistic regression, random forest, support vector machine (SVM), artificial neural network, Gaussian naive Bayes); Used NLP (latent Dirichlet allocation - LDA) for free-text notes. Experiment 2: multilabel classification model (convolutional neural network). | A broad range of 22 SDoH categories (see page 5). | The authors tested multiple methods of expanding or collapsing data points based on whether encounters were a data point or a patient. The DuPage County study (Experiment 2) included data from 435 participants; used 274 unique patient data points for Chinatown dataset. | Experiment 1: random forest classifier had the best accuracy and overall accuracy to predict SDoH: 71% using demographic and navigator data. Experiment 2: greatest accuracy was 73% solely using demographic and augmented data. |
| Schario et al., 2022 | U.S. | To explore nurse care management experiences with chatbots and provide insight on implementation. | Description of patient cases | ACO in university hospitals of Cleveland, Ohio | Case study | Addressing unmet social needs through care coordination, integration, or care management. | NLP | AI chatbot. Care manager receives alerts and documentation of chatbot conversations when needed, to assist the care manager with providing tailored intervention based on their needs. | Mentions generally that SDoH can be better addressed through chatbot communication. Discussed education, referrals to social work, tobacco cessation education, and others. | NA - two cases | The use of chatbots have assisted with removing barriers in care and facilitated relationships and communication between patients and care managers. |
| Bako et al., 2021 | U.S. | To parse and classify events where social workers addressed patients' unmet social needs using ML and NLP. | Existing patient data from EHR (clinical notes, demographics, clinical characteristics, social worker notes). | Primary care: Eskenazi Health (safety net provider with hospital and FQHC) in Indianapolis | Retrospective analysis | Social resource provision or indirect identification of unmet social needs. | NLP, ML | NLP, ML/deep learning to automatically classify events where social workers intervened on patients' unmet social needs. Tested multiple algorithms: rule-based, logistic regression, kernelized SVM, linear SVM, and Multinomial Naive Bayes. | Classification scheme of social work interventions included financial planning, supportive counseling, care coordination, education, community service, application filing and reporting, housing, transportation, durable medical equipment, or legal services. | 408 adults, 815 social work events | Improved categorization in ML/deep learning compared to rule-based models, with high accuracy: kernelized SVM (0.97), logistic regression (0.96), linear SVM (0.95), Multinomial Naive Bayes (0.92). Three quarters (73%) of social worker clinical notes indicated at minimum one intervention, particularly care coordination (21.5%), education (21%), financial planning, community resource referrals (17%), counseling (15%). |
| Bako et al., 2020 | U.S. | Identify the unmet social needs of patients referred to social work, their sociodemographic and medical characteristics, and common co-occurring unmet social needs. | EHR and local health information exchange system database (Indiana Network for Patient Care) | Eskenazi Health outpatient primary care clinics in Indianapolis | Secondary analysis of a longitudinal cohort study | Social resource provision or indirect identification of unmet social needs. | NLP, ML | NLP and market basket analysis (examine patterns related to co-occurring products; specifically, to understand the order of unmet social needs that led to a social worker referral). | 17 social needs categories were used based on literature search, and social work categories included financial, food, violence/safety, housing, legal, transportation, behavioural health, aging, education, employment, family/social support, pregnancy, language services, disability, community resources, adherence to medication/treatment. | All adult patients (33,683), patients not receiving social worker referral (26,355) and those who do (7,328). | - 22% of all adult patients were given a social worker referral. - 25% of referrals were financial related or pregnancy-related  - Most common co-occurring needs: 7% were pregnant and had difficulties with English language, and 3% had family and social support challenges and behavioural health issues. |
| Gray et al., 2023 | U.S. | Create and evaluate a rule-based NLP and text mining model that can characterize residential instability, transportation needs, and food insecurity from EHR data. | EHR data | Maryland, Johns Hopkins Health System | Retrospective analysis/ chart review | Identify or predict unmet social needs | NLP, ML | NLP and text mining. Rule-based and deep learning ML models (note-based, terminology-based, and ClinicalBERT). | Residential instability, food insecurity, transportation challenges. | 1,317,335 adult patients with Maryland code for matching; validation dataset of 192 participants. | Rule-based models: residential instability, particularly homelessness performed the best: weighted avg. precision: .92, recall: .84, F1: .92; and housing insecurity: .84, .82, .79. Food insecurity performance was lower, and transportation challenges had the lowest performance. |
| Hatef et al., 2022 | U.S. | Examine whether a rule-based NLP algorithm could accurately identify residential instability from unstructured EHR data across three integrated health systems. | EHR across three health systems | Johns Hopkins Health Systems (JHHS), Kaiser Permanente Mid-Atlantic States (KPMAS), Kaiser Permanente Southern California (KPSC) | Retrospective analysis/ chart review | Identify or predict unmet social needs | NLP | Rule-based NLP system that was informed by experts in medicine and residential instability. | Residential instability (homelessness and housing instability) | 3572 (JHHS); 8197 (KPMAS); 300 (KPSC) | The algorithm had moderate results: precision across the sites (range: 0.45-1.0), sensitivity (0.68-0.96) and specificity (0.69-1.0). The authors conclude that that their NLP algorithm performs across healthcare organizations to identify unmet social needs from EHR data. |

ACO: Accountable Care Organization

EHR: Electronic health record

FQHC: Federally Qualified Health Centers

HIPAA: Health Insurance Portability and Accountability Act

ML: Machine learning

NLP: Natural language processing

SDoH: Social determinants of health
